# Evaluation of mobile learning: Students' experiences in a new rural-based medical school

**DOI:** 10.1186/1472-6920-10-57

**Published:** 2010-08-11

**Authors:** Debra Nestel, Andre Ng, Katherine Gray, Robyn Hill, Elmer Villanueva, George Kotsanas, Andrew Oaten, Chris Browne

**Affiliations:** 1 Gippsland Medical School, Monash University, Gippsland Campus, Northways Road, Churchill, Victoria, Australia; 2 Medicine Nursing and Health Science, Monash University, Clayton Campus, Wellington Road, Clayton, Victoria, Australia

## Abstract

**Background:**

Mobile learning (ML) is an emerging educational method with success dependent on many factors including the ML device, physical infrastructure and user characteristics. At Gippsland Medical School (GMS), students are given a laptop at the commencement of their four-year degree. We evaluated the educational impact of the ML program from students' perspectives.

**Methods:**

Questionnaires and individual interviews explored students' experiences of ML. All students were invited to complete questionnaires. Convenience sampling was used for interviews. Quantitative data was entered to SPSS 17.0 and descriptive statistics computed. Free text comments from questionnaires and transcriptions of interviews were thematically analysed.

**Results:**

Fifty students completed the questionnaire (response rate 88%). Six students participated in interviews. More than half the students owned a laptop prior to commencing studies, would recommend the laptop and took the laptop to GMS daily. Modal daily use of laptops was four hours. Most frequent use was for access to the internet and email while the most frequently used applications were Microsoft Word and PowerPoint. Students appreciated the laptops for several reasons. The reduced financial burden was valued. Students were largely satisfied with the laptop specifications. Design elements of teaching spaces limited functionality. Although students valued aspects of the virtual learning environment (VLE), they also made many suggestions for improvement.

**Conclusions:**

Students reported many educational benefits from school provision of laptops. In particular, the quick and easy access to electronic educational resources as and when they were needed. Improved design of physical facilities would enhance laptop use together with a more logical layout of the VLE, new computer-based resources and activities promoting interaction.

## Background

In this paper we describe the evaluation of a mobile learning (ML) project in a new rural-based medical school at Monash University, Australia. The literature contains many definitions of ML [1]. For example:

*"The intersection of mobile computing and e-learning: accessible resources wherever you are, strong search capabilities, rich interaction, powerful support for effective learning, and performance-based assessment. E-learning is independent of location in time or space."*[2]

ML potentially has many advantages such as increasing access to resources when and where they are needed and connecting learners with each other and faculty [3,4]. There are two core elements, first, the hardware and the second, the virtual learning environment (VLE). Several authors identify ways in which ML facilitates collaborative learning with time efficient sharing of materials and feedback [5-8]. Electronic textbooks and other reference materials replace hardcopy. Content is available 'just in time' and can include audiovisual materials. Creatively designed learning resources are interactive and enjoyable motivating learners. Orientation for users is recommended [3,9,10] together with appropriate fall back solutions should the technology fail [11]. Information technology support is essential for success. Tutors have reported the value of ML incorporated into curricula, as an adjunct to other educational methods and as a means of engaging otherwise disinterested learners [1].

Challenges with ML include assessment, supporting learning across contexts and settings, ensuring 'proper' use of personal and professional content. ML challenges both students and faculty to develop their technological proficiencies in order to take maximise the benefits [9,11,12]. Several factors are likely to influence the effectiveness of ML. These include hardware, software, physical infrastructure; and, student and faculty preparedness.

### Mobile learning in medical education

Although there are some examples in the medical and health care professional literature on the role of ML, most focus on a particular element of support (e.g. clinical log books) or content (e.g. clinical information) rather than a broad spectrum of activities. Further, most examples relate to highly mobile handheld computers rather than laptops. The latter have distinct advantages over other mobile devices due to the larger visual field, enhanced functionality and capability in the provision and capture of information. However, they are also larger and weigh more so are less portable.

Compared with traditional educational methods, ML in medical (and veterinary) education has demonstrated at least similar outcomes [3,4,11,13]. VLEs can facilitate a transition from teacher- to learner-centred education [14]. Synchronous interactive communication sessions are also valued [10,13,15]. However, faculty need to develop skills to coordinate these sessions and troubleshoot technology across distributed sites [16,17].

### Mobile learning technologies

Laptops, personal digital assistants and tablet personal computers have been shown to be effective and feasible mobile technologies in medical education providing access to the internet, software and information repositories which can enhance students' learning experiences [18,19]. Sharing information within and between cohorts can increase the efficiency and effectiveness of educational programmes [20].

### Mobile learning at Gippsland Medical School

Gippsland Medical School (GMS) offers a graduate entry medical programme. Students largely spend first year on campus while the remaining three years are almost entirely in clinical settings in outer metropolitan and rural Victoria, Australia. All students are issued with a laptop at the commencement of their studies. The laptop remains the property of the medical school with students taking a custodial role. A key driver for this initiative was to facilitate student access to curriculum materials and licensed software both on campus and across a broad geographical area.

Students were issued with a Hewlett Packard 6910p laptop. The specifications met the anticipated needs of students. The total cost of purchase was AU$72,960 for the first cohort of medical students. For the VLE, we used a customised platform, Monash University Study Online (MUSO), which contains two main teaching and learning tools, Blackboard and Interlearn, for course management and facilitation of interaction online. Table 1 lists the licensed software made available for students on the laptops.

**Table 1:** Licensed software made available on student laptops

Productivity software	Microsoft Office
	Endnote
	Toolbook Runtime (with modules)
**Medical Software (Anatomy)**	A.D.A.M
	Anatomy TV
	Grant's Atlas of Dynamic Human Anatomy
	Netter
	Imaging Modalities
	D.A.R.T.O.S.

**Medical Software (Biochemistry)**	Buffers
	Clustal X
	Cn3D
	Genedoc
	Graphpad Prism
	Growcyt
	Hypercell 98
	Image J
	Interactive biochemistry
	NjPlot
	Protein explorer
	Protein purification lab
	RasMol 2.6
	Spectrophotmetry
	Swiss PDB Viewer
	Trace

**Medical Software (Nutrition and Dietetics)**	FoodWorks
	SERV v5.5e

**Medical Software (Pharmacology)**	Pharma-Cal-Ogy

**Medical Software (Physiology)**	Acid Base
	Exercise Physiology

The first cohort of fifty-seven students commenced the four-year Bachelor of Medicine and Bachelor of Surgery degree in 2008. There were 37 female and twenty male students, aged 22 to 44 years (mean = 26). Most students came from Victoria while six were international students. Nine students were classified as having a rural background. Most students had a biological, medical, health or applied science degree (68%). Remaining students (20%) held health professional degrees in nursing, pharmacy, physiotherapy or other degrees (12%).

The research questions explore students' experiences of ML:

1. In what ways does ML support learning?

2. What areas need development?

## Methods

These questions were addressed using quantitative and qualitative research methods. A questionnaire (Additional file 1) and individual interviews with students were designed to explore students' experiences of using laptops and ML. All students were invited to complete the questionnaire. Quantitative data were entered to SPSS 17.0 and descriptive statistics computed. Free text comments were entered to Microsoft Word and analysed thematically. Convenience sampling was used to select students who participated in individual interviews. Topic guides for interviews were developed (Additional file 2). Interviews were audiotaped and thematically analysed. The Standing Committee on Ethics in Research Involving Humans approved the study (CF08/0906-2008000429).

## Results

### Questionnaire

Fifty students responded to the questionnaire representing 88% of the cohort. Twenty-eight students (56%) reported prior ownership of a laptop. Students' use ranged from one to fifteen hours a day with four hours being the modal daily use (28%). Twenty-six students (52%) reported bringing their laptop to GMS everyday while the remaining students brought their laptops up to three times weekly. Twenty-seven students (54%) would recommend this laptop.

In terms of managing laptops, twelve students (24%) reported that they did not back up their work. Other students reported backing up content daily (2%), weekly (36%) or monthly (24%).

Students reported using the laptops for wide ranging activities including all the programs for which licensed software was obtained. Most frequent use was for the internet (98%), email (98%), word processing (94%), presentations (94%) and library resources (92%). Almost all students would like access to the internet wherever they go outside the university. The most frequently used applications were Microsoft Word (98%) and PowerPoint (90%). Less frequently used applications included Excel (52%) and bibliographic software (38%). Students requested additional applications such as iTunes (70%), Skype (50%) and MindManager (22%).

We established an arbitrary score for high use and high satisfaction (> 4.5) for student reporting on MUSO. Table 2 shows students' highest regular use of MUSO for curriculum content (e.g. lecture, tutorial and practical notes), student information (e.g. announcements and updated timetables) and formative and summative assessments. Table 3 shows students' highest satisfaction with MUSO items that they used most regularly. Assessments received the highest mean scores followed by materials for problem-based learning and operational information.

**Table 2:** Students' ratings of frequency of use of the virtual learning environment from *'never use' *(1) to *'always use' *(6) (n = 50)

	Mean	SD	Min	Max
Lecture notes	5.8	0.6	3	6

Tutorial notes	5.4	1.2	1	6

Practical notes	5.4	1.2	1	6

Announcements	5.4	1.1	2	6

Summative assessment (Exams)	5.4	1.0	1	6

Updated timetables	5.2	1.1	2	6

Formative assessment (Quizzes; Assignments)	5.2	1.1	2	6

Community Based Placement Program information	4.6	1.3	1	6

Problem-based learning materials	4.0	1.7	1	6

External weblinks	3.8	1.6	1	6

Overviews	3.7	1.8	1	6

Applications	3.7	1.5	1	6

Weblinks	3.5	1.5	1	6

**Table 3:** Students' ratings of satisfaction with various aspects of the virtual learning environment from *not at all *(1) to *completely satisfied *(6) (n = 50)

	Mean	SD	Min	Max
Summative assessment (Exams)	5.0	1.2	2	6

Formative assessment (Quizzes; Assignments)	4.8	1.1	2	6

Updated timetables	4.7	1.2	2	6

Problem-based learning materials	4.6	1.2	2	6

Lecture notes	4.5	1.1	2	6

Overviews	4.4	1.3	2	6

Announcements	4.4	1.5	1	6

Community based Practice Program information	4.4	1.4	2	6

Tutorial notes	4.3	1.0	2	6

Practical notes	4.3	1.1	2	6

Applications	4.2	1.3	1	6

Weblinks	4.0	1.4	2	6

External weblinks	3.8	1.6	1	6

Thirty-eight students (76%) thought that MUSO could be improved. Suggestions included greater clarity in the organisation of content with a system for prioritising critical and key information posted by GMS staff. Reducing the number of clicks to get to desired information would be helpful. Direct access to lecture notes from clicking on the timetable would be valuable.

Students rated their satisfaction with the technical support for the programme on a 3-point scale from not at all (1) to completely (3) satisfied. Sixty-five percent of students were partially or completely satisfied. In free text comments students praised the GMS IT support but made several suggestions to improve campus IT support including greater listening and explaining skills from University and faster turnaround times.

### Individual interviews with students

Six students participated in individual interviews lasting between seventeen and 23 minutes. Interviews were reported to be cordial and perceived to be honest, with participants speaking confidently and frankly. Emergent themes related to students' responses to being given laptops, perception of learning supported by laptops, laptop specifications, management of laptops and MUSO.

### Responses to being given laptops

Students thought the provision of laptops was generous and they were very grateful for this support. All mentioned the reduced financial burden. One student was nervous since s/he lacked confidence in computer skills.

"I was extremely grateful. It is something I couldn't afford and didn't expect." S2

"I was excited and happy. It was unexpected. It was hugely beneficial from a financial perspective. Also the fact that it has everything required for the course. That is brilliant and generous." S4

### Perception of learning supported by laptops

Students reported the laptops enhanced learning by providing an equitable and accessible platform for seeking, recording and exchanging information, gave options for cognitive processing of information, were core to the curriculum and portable. The ability to use laptops in lectures and tutorials was valued. However, some students noted open laptops created barriers in lectures.

"I have made summaries on my own laptop in the past and having to do everything myself is good. Nowadays it is easy to just cut and paste images etc... so I am getting it done but not learning as I would if I was reading the textbook. You have to have more than one way to learn to do well in this course..." S2

"... because of the laptop I can do a lot of my own research, I often have my laptop open during the lectures to do my own research, I do all my notes. I have all my music and stuff because I use it for a lot of my own personal stuff. I use it a lot. If it wasn't for the laptop I wouldn't do half the study." S3

"They are huge. Having all the programs. Like A.D.A.M, other anatomy ones. Also having a laptop. I have never had one before. They are very mobile. As everybody's are equal they are consistent. We can send things to each other and know it will be okay." S4

"The mobility. Taking information with you, and the resources are equal, everybody has the same." S6

### Laptop specifications

In response to being asked about expectations of the laptop, students identified a strong, robust, compact, portable, light laptop with wireless capability and sufficient memory.

### Management of laptops

One student thought that as graduates they should have more responsibility over content management of their laptops.

"However we are limited to the rights. We can't even move the icons on the desktop. It is irritating but its okay. I wanted to play a particular disk that comes with one of the books I bought but I couldn't because I was restricted." S2

Problems reported included limited capacity to save work because of insufficient memory (RAM and/or hard drive capacity), the internet search engine (Firefox), short battery life and too few power sockets in teaching spaces. Students resented returning their laptops for re-imaging. That is, restoring the laptop to its default state.

"The unreliability. I have had a few instances where I have had to re-image the laptop which has meant I have to set up printers again and redo notes. It can take a lot of time. And be frustrating." S6

### The virtual learning environment - MUSO

Students made several comments about MUSO. They were positive about being able to access notes, lecture slides and readings prior to lectures and to audio taped lectures.

"...it is a very handy resource, it saves me a lot of time. I get the notes easily, I use it everyday." S2

"I like that all the files are available for downloading and viewing of lectures and readings. Having all the assessment (practice and proper) and key dates available. The blogs and forms I don't use though. I don't think they are useful." S3

"It has been really good. In the first semester I struggled but I have improved with my usage though. But having to check all the different folders for things is difficult. It would be easier if you could get it all in one. It is more contained and organised this semester. I haven't had any issues with it this semester. It's good to have the lecture notes prior to the lecture but that's up to the lecturers. Having the lectures taped is also good. At times I haven't been able to make it and knowing that you can listen to it is good. " S4

"I like the folders divided into semesters and the communications folder with the announcements." S5

Some students were dissatisfied with the layout of information, sequencing, opening documents, down loading files, too much change.

"...its a lot of clicking, but it is expected because of the sheer volume that everything is recorded on." S1

"I don't really like it, sometimes it can be a pain. But you have to use it to get the things you need. If I need to see announcements that's the easier place to do it. Also the fact that you can't download a lot of things from it. The Firefox blocks a lot of it." S1

"In one word, evolving. It changes so much. The file structure and the folders and the naming of files, if you blink it changes. The new format that the lectures open in makes it harder for them to save. It would be nice for it to be organised and constant." S3

"I find it both useful and dysfunctional. Although it has a lot of information, sometimes it can be inefficient. It's not bad. It just seems a bit clumsy." S6

Students suggested improvements to MUSO that included improved orientation at the beginning of term, improved functionality permitting students to download files more efficiently and enabling "*going back*" and an improved structure.

"...I don't feel like there was sufficient time and effort put into familiarising the students with MUSO." S2

"I just wish I could download all the lectures in one go. It seems like it could be done more efficiently from a students' perspective." S2

"...being able to download everything for the week and an online timeline of what you have combine up and what's due in and holidays, a bit of a calendar would be great.? ... like on the tasks page on outlook. Something that just shows all the important GMS dates." S2

*"Perhaps a network system that saw all Monash campuses linked. For example, it would be nice to see what medicine is doing in Melbourne, Malaysia, undergraduate..." S*6

## Discussion

Students were positive about the laptop programme supporting their learning. There were features of the hardware and the VLE that supported learning while there are also areas for development. School issued laptops also reduced financial pressure. Educational benefits included access to resources when and where the students needed them, especially during structured curriculum activities such as lectures and tutorials (e.g. problem-based learning sessions). Being able to create and personalise lecture notes was beneficial. MUSO provided a satisfactory environment for supporting students in these activities.

Providing the same laptop ensured that all students had equitable access to resources. The mobility of the laptop meant students could access resources in a variety of locations. This enabled students to undertake their curriculum and importantly for this group of adult learners allows them to manage their own learning resources.

Preferences for electronic resources by the current generation of higher education students are reported [21-23]. Advantages of electronic resources include access speed and ease of use, both of which are supported in our study.

Several challenges were associated with laptops as an effective ML tool. The design of lecture theatres did not readily accommodate all students using laptops. Power sockets and wireless connectivity were inadequate. Students reported that laptops provided a physical barrier when used in lectures but this was not noted in tutorials. Some students reported the battery life of the laptop was poor and there was limited capacity to save work due to insufficient memory.

Although highly valued by students, ML at GMS needs improvement. The areas for development include the electronic learning resources and MUSO. Students requested more materials, especially audiovisual resources, a more logically arranged virtual learning environment and more flexibility in accessing materials.

Students' suggestions for improvements in the ML project have been addressed in several ways. These include school issued guidelines on managing laptops (e.g. backing up data and orientation to basic hardware and software), improvements to MUSO (e.g. steam lining organisation of information reducing the number of clicks to access resources and orientation for students) and the provision of power boards in teaching spaces to address poor battery life.

## Limitations of the study

The broader application of these findings is limited by our single cohort in one medical school and that we are a new program. Students who responded to the questionnaire may not have represented the views of the entire cohort. Similarly, students who participated in interviews did not necessarily reflect the views of their colleagues. Although students overall offered strong support for ML and students indicated that it enhanced learning we do not know if perception is reality. We did not link demographic variable with results so do not know if there are age and sex differences in response to ML. It is difficult to isolate variables in ML that make it effective. It is only as good as the resources within. Further areas for study

In this study we were only able to evaluate the students' use of ML between campus and home. In subsequent years of the medical degree, students will take their laptops into clinical settings across the region further testing the potential of ML and addressing a key driver for the program. In order to gain a broader perspective of ML we intend to explore faculty responses to ML, their knowledge and perception of its' potential and consider ways to incorporate ML with other educational methods.

## Conclusions

Students in this study reported educational benefits of ML. The provision of a laptop by the school facilitates timely and easy access to a range of educational resources and electronic materials. The VLE supported learning by offering a variety of educational resources accessible whenever students required them. Some areas of the ML program need further development including the design of physical infrastructure of the medical school and improvements to the VLE.

## List of Abbreviations

A ML: Mobile Learning; GMS: Gippsland Medical School; VLE: Virtual Learning Environment; MUSO: Monash University Study Online.

## Competing interests

The authors declare that they have no competing interests.

## Authors' contributions

DN was involved in developing the evaluation strategy for the project, conducting thematic analysis on the data and drafting and writing the manuscript. AN was involved in conducting student interviews and thematic analysis of the data. KG was involved in drafting and writing the manuscript. AO was involved in data entry and thematic analysis. AN, RH, EV, GK, AO and CB were all involved in the project implementation. All authors read and approved the final manuscript.

## Pre-publication history

The pre-publication history for this paper can be accessed here:

http://www.biomedcentral.com/1472-6920/10/57/prepub

## Supplementary Material

Additional file 1 **Questionnaire**. The questionnaire that was used in our study Click here for file

Additional file 2 **Topic guide for interviews**. The topic guide that facilitators used in the focus groups Click here for file
