# Supplementary material for: Evaluation of mobile learning: Students' experiences in a new rural-based medical school
Source: BMC Med Educ. 2010 Aug 11;10:57. doi: 10.1186/1472-6920-10-57 (PMC2928245; doi:10.1186/1472-6920-10-57)
Supplement: Additional file 1 — Questionnaire. The questionnaire that was used in our study [file 1472-6920-10-57-S1.PDF]

Laptops

1. Did you have your own laptop before you started at GMS? Yes/No
2. On average, how many hours do you use your laptop each day? _______ hours
3. Do you bring your laptop to GMS everyday? If not, how many times per week?
4. Which of the following applications are you using on your laptop?
    a. Microsoft Word yes/no
    b. Microsoft Powerpoint yes/no
    c. Excel yes/no
    d. Reference Manager yes/no
    e. EndNote yes/no
    f. Skype yes/no
    g. Other yes/no

    Please list:

5. What applications do you want to use on your laptop?

Please list:

6. Would you recommend this HP laptop? yes/no

Please briefly explain your answer.

7. What activities do you use your laptop for?
    a. Access to library resources yes/no
    b. Access to the internet yes/no
    c. Email yes/no
    d. Using multimedia resources (CDs, books etc) yes/no
    e. Using multimedia resources from the internet? yes/no
    f. Preparing presentations yes/no
    g. Word processing yes/no
    h. Other yes/no

    Please specify:

8. What applications that you think would enhance your learning would you like installed on your computer?
   a. iTunes yes/no
   b. Skype yes/no
   c. MindManager yes/no
   d. Other yes/no

   Please specify

9. How often do you back up your work on your laptop? (Circle one)
   a. NEVER
   b. DAILY
   c. WEEKLY
   d. MONTHLY
   e. OTHER, please specify ______________________________

10. Would you like to have access to the internet wherever you go (Outside Monash University too)? yes/no

11. How satisfied are you with the technical support you have received for your laptop? (Circle one)
    a. Not at all satisfied
    b. Partially satisfied
    c. Completely satisfied

12. What could we do to improve technical support?

13. To what extent do you think it will be helpful to have your laptop in Year B?
    a. Not at all helpful
    b. Partially helpful
    c. Completely helpful

The next questions relate to MUSO. We would like you to rate how often you use MUSO and then your satisfaction

14. MUSO is divided into several sections. Rate the degree to which you use each section.

	Never			All the time		
**Session notes**						
Overviews	1	2	3	4	5	6
Lecture notes	1	2	3	4	5	6
Tutorial notes	1	2	3	4	5	6
Practical notes	1	2	3	4	5	6
PBL materials	1	2	3	4	5	6
Applications	1	2	3	4	5	6
Weblinks	1	2	3	4	5	6
**Student information**						
Announcements	1	2	3	4	5	6
Updated timetables	1	2	3	4	5	6
CBPP information	1	2	3	4	5	6
**Learning activities**						
Formative assessment (Quizzes; Assignments)	1	2	3	4	5	6
Summative assessment (Exams)	1	2	3	4	5	6
**External weblinks**	1	2	3	4	5	6

15. How satisfied are you with each section.

	Not at all satisfied			Completely satisfied		
**Session notes**						
Overviews	1	2	3	4	5	6
Lecture notes	1	2	3	4	5	6
Tutorial notes	1	2	3	4	5	6
Practical notes	1	2	3	4	5	6
PBL materials	1	2	3	4	5	6
Applications	1	2	3	4	5	6
Weblinks	1	2	3	4	5	6
**Student information**						
Announcements	1	2	3	4	5	6
Updated timetables	1	2	3	4	5	6
CBPP information	1	2	3	4	5	6
**Learning activities**						
Formative assessments (Quizzes; Assignments)	1	2	3	4	5	6
Summative assessments (Exams)	1	2	3	4	5	6
**External weblinks**	1	2	3	4	5	6

16. How do you think we can improve MUSO?
