# Supplementary material for: Evaluation of mobile learning: Students' experiences in a new rural-based medical school
Source: BMC Med Educ. 2010 Aug 11;10:57. doi: 10.1186/1472-6920-10-57 (PMC2928245; doi:10.1186/1472-6920-10-57)
Supplement: Additional file 2 — Topic guide for interviews. The topic guide that facilitators used in the focus groups [file 1472-6920-10-57-S2.PDF]

## Additional file 2 - Topic guide for interviews

1. What was your initial response to receiving a laptop as an integral part of your learning?
2. What expectations did you have of the laptops?
3. To what extent have the laptops met your expectations?
4. What advantages are associated with having a GMS laptop?
5. What are the disadvantages?
6. Any other comments?
7. Overall, what are your impressions of MUSO?
8. How often do you use MUSO?
9. What do you like about MUSO?
10. What do you dislike about MUSO?
11. In what ways can MUSO be improved?
12. Any other comments?
